# Recruitment, Growth and Mortality of an Antarctic Hexactinellid Sponge, *Anoxycalyx joubini*

**DOI:** 10.1371/journal.pone.0056939

**Published:** 2013-02-27

**Authors:** Paul K. Dayton, Stacy Kim, Shannon C. Jarrell, John S. Oliver, Kamille Hammerstrom, Jennifer L. Fisher, Kevin O’Connor, Julie S. Barber, Gordon Robilliard, James Barry, Andrew R. Thurber, Kathy Conlan

**Affiliations:** 1 Scripps Institution of Oceanography, La Jolla, California, United States of America; 2 Moss Landing Marine Laboratories, Moss Landing, California, United States of America; 3 Hatfield Marine Science Center, Newport, Oregon, United States of America; 4 Swinomish Indian Tribal Community, La Conner, Washington, United States of America; 5 Cardno Entrix, Gig Harbor, Washington State, United States of America; 6 Monterey Bay Aquarium Research Institute, Moss Landing, California, United States of America; 7 College of Earth, Ocean, and Atmospheric Sciences, Oregon State University, Corvallis, Oregon, United States of America; 8 Canadian Museum of Nature, Ottawa, Ontario, Canada; Heriot-Watt University, United Kingdom

## Abstract

Polar ecosystems are sensitive to climate forcing, and we often lack baselines to evaluate changes. Here we report a nearly 50-year study in which a sudden shift in the population dynamics of an ecologically important, structure-forming hexactinellid sponge, *Anoxycalyx joubini* was observed. This is the largest Antarctic sponge, with individuals growing over two meters tall. In order to investigate life history characteristics of Antarctic marine invertebrates, artificial substrata were deployed at a number of sites in the southern portion of the Ross Sea between 1967 and 1975. Over a 22-year period, no growth or settlement was recorded for *A. joubini* on these substrata; however, in 2004 and 2010, *A*. *joubini* was observed to have settled and grown to large sizes on some but not all artificial substrata. This single settlement and growth event correlates with a region-wide shift in phytoplankton productivity driven by the calving of a massive iceberg. We also report almost complete mortality of large sponges followed over 40 years. Given our warming global climate, similar system-wide changes are expected in the future.

## Introduction

**Figure 1:**
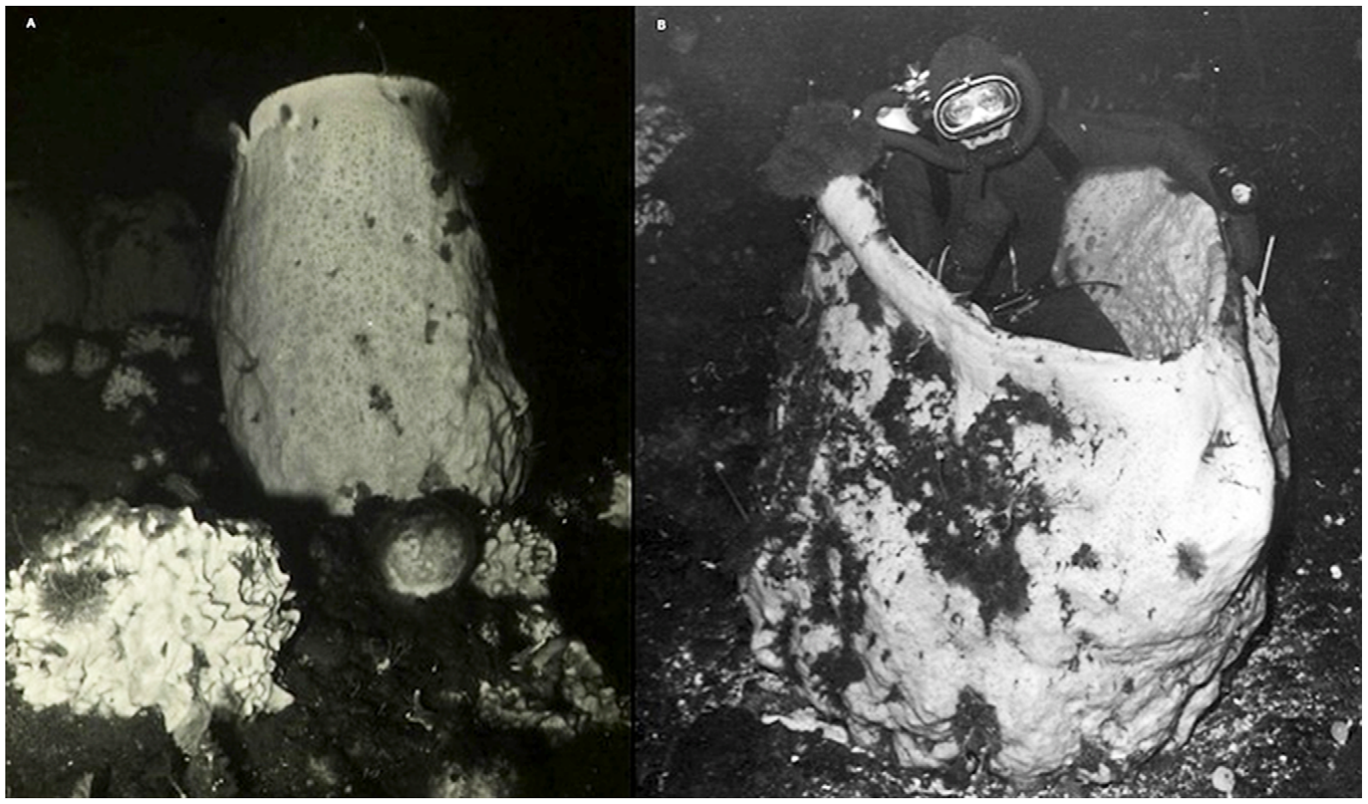
Large *Anoxycalyx joubini* at Cape Armitage, McMurdo Station. Two large *A. joubini* at a depth of 60 m, 1967. (A) The larger sponge on the right (*A. joubini*) is approximately 2 m tall. (B) *A. joubini* in photo is approximately 1.75 m tall.

The ancient hexactinellid sponges are associated with the Ediacaran Period, and they are one of the basal metazoans [1], [2]. Despite great interest in the group, the natural history of the Hexactinellida is poorly known, and the life history is largely derived from inferences. The Antarctic hexactinellid fauna is well described [3]–[7]; however, they are generally found at depths greater than 30 m, making it very difficult to study them *in situ*. Thus, life history patterns of recruitment, growth and reproduction are poorly understood for most hexactinellid sponges, although they have been thought to be very slow in comparison to the more common demosponges. Much of this characterization of low recruitment and growth rates of Antarctic sponges is based on early research at McMurdo Sound [2], [8]–[10]. Three species [11] of hexactinellid sponges comprise the bulk of the benthic biomass: the relatively small *Rossella antarctica* is very common, reproduces by budding and exhibited growth during the study, while the massive, volcano-shaped hexactinellids, *Anoxycalyx (Scolymastra) joubini* and *Rossella nuda/racovitzae* [11], had no recruitment or growth [9]. *Anoxycalyx joubini* is the largest and most conspicuous sponge in the Antarctic and although it has been observed as much as 2 m in height (Figure 1), it has never been observed to settle or grow which has led to estimates of extreme longevity [7], [10].

Here we present observations of remarkable episodic recruitment and growth and apparently high mortality of the hexactinellid, *A. joubini*, in McMurdo Sound, Antarctica. These observations call into question the validity of previously held generalizations, at least for this conspicuous species.

## Methods

**Figure 2:**
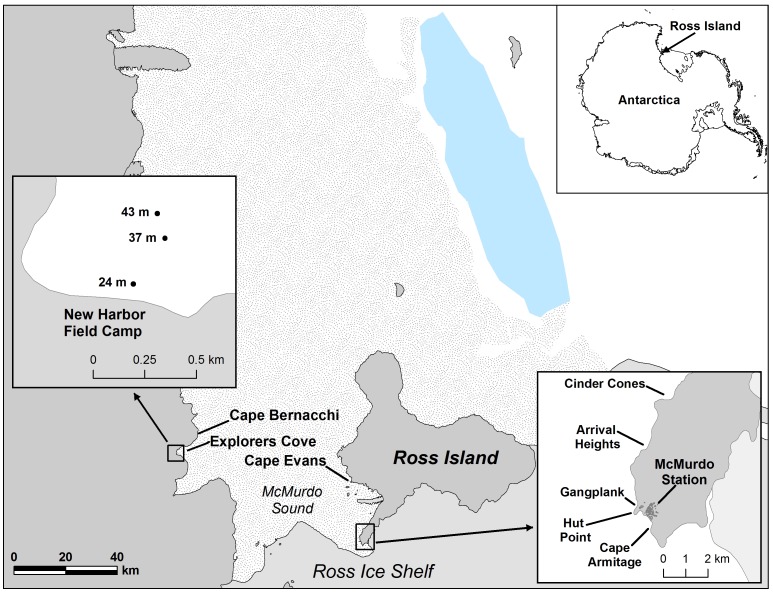
Map of McMurdo Sound, Antarctica, with the sites discussed in this paper. The blue rendering of iceberg B 15 in 2004 is in the upper right above Ross Island on the map.

The observations of recruitment and growth occurred on artificial, experimental structures located on each side of McMurdo Sound (Figure 2). The area around McMurdo Station is normally influenced by southerly currents bringing phytoplankton from a region north of Ross Island, which is often dominated by a large and productive polyna. Slow northerly currents sourced beneath the Barrier Ice bathe the Explorers Cove region of New Harbor. The currents at Explorers Cove advect very different and minimal plankton because the water mass has circulated under the Ross Ice Shelf [12], [13].

With the exception of a wooden gangplank dropped from a ship in 1960, all structures were purposefully placed in the 1960s and early 1970s. The gangplank is at a depth of 25–30 m located just north of Hut Point, McMurdo Station (Figure 2). During the 1970s, the gangplank and the surrounding area were subject to massive settlement of the demosponge, *Homaxinella balfourensis*; however, essentially all of these sponges were removed by anchor ice in the mid-1980s [14], [15]. Various cages in the vicinity of McMurdo Station were placed in 1967. Settling surfaces either supported off the substratum by iron and wood posts or suspended beneath floats as much as 30 m off the bottom were established along the Hut Point Peninsula from Cape Armitage to Cape Evans in 1974.

Various types of settling substrates composed of PVC plates and pipes were suspended in the water column by floats at Explorers Cove in 1974–75. These floaters, or settling structures suspended by floats up to 20 m above the floor were at bottom depths of 24–43 m (Figure 2). In addition, platforms placed in the 1970s for experiments [16] also served as habitat for recruitment and growth of sponges.

This paper is based on regular observations from 1967 at Ross Island sites and from 1974 at Explorers Cove through the end of 1989. The Ross Island sites were re-visited in 1998, and all sites were re-visited in 2004. In 2004, photographs were taken of *A. joubini* that had settled on the gangplank and on Explorers Cove structures. Detailed sponge photographs were obtained in 2010 and one site was revisited and photographed in 2012. While the specific sites discussed in this paper were not visited between 1989 and 2004, no recruitment of the very conspicuous *A. joubini* was observed in the late 1990s on Ross Island. Therefore it is reasonable to assume that during the extensive diving by Kathy Conlan and others in that period, there would have been observations of some new sponges, had there been a strong recruitment event much prior to 1998.

Imagery data were collected using a micro-ROV and diver photographs. The sponges were measured using parallel lasers on cameras, 20 cm apart on the camera and 10 cm on the ROV. We did not collect the sponges, but estimated the mass from a regression published in Dayton *et al*., (1974) [8]: Y = 0.348x^2.880^; Y = wet weight (g), and x = maximum diameter of sponge (cm). This equation was generated in 1968 when 102 sponges were collected, measured, and weighed. At that time, two species of sponge, *A. joubini* and *R. nuda/racovitzae,* were lumped together [8]. Most *R. nuda/racovitzae* are smaller than *A. joubini*, but *R. nuda/racovitzae* is likely heavier than the similar-sized *A. joubini* because the latter is characterized by a larger central cavity. Considering this, the biomass estimates of *A. joubini* derived from the equation are probably high, but are the best possible with the data on hand and they are consistent within this study.

### Ethics Statement

No permits were required for this study and the region is covered by the Antarctic Treaty so it does not involve private property. No protected species were involved with the study and no animals were husbanded. We have the following interests: Gordon Robilliard is employed by Cardno Entrix. This does not alter our adherence to all the PLoS ONE policies on sharing data and materials, as detailed online in the guide for authors.

## Results

**Figure 3:**
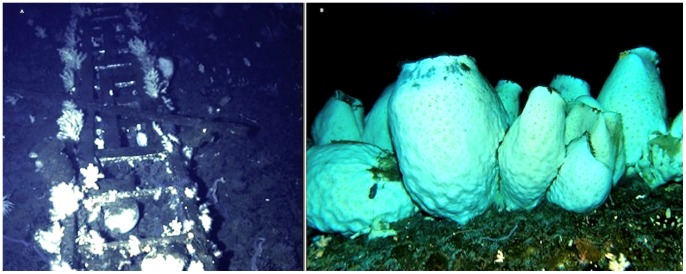
*Anoxycalyx joubini* on the gangplank at Hut Point, McMurdo Station. (A) 1989, after anchor ice had removed 100% cover of large *H. balfourensis* [15], (B) the same gangplank with *A. joubini* in 2010.

There was no sponge colonization on the gangplank in the 1960s, but in 1974–1978 there was virtually no anchor ice formation [14], facilitating heavy recruitment and settlement of the demosponge, *H. balfourensis* over the entire area [12]. Anchor ice returned in the 1980s removing *H. balfourensis* [15], and by 1989 the gangplank was clean of sponges (Figure 3). From 1967 through 1989, there were no hexactinellid sponges on the gangplank, but sometime between 1989 and 2004, *A. joubini* settled and grew there. In 2010, 19 *A. joubini* were photographed on the gangplank, which together had an estimated mean biomass of 30 kg, with the largest sponge weighing over 76 kg (Figure 3). While several new *A. joubini* were observed on the bottom in the vicinity of McMurdo Station, only one other *A*. j*oubini* had settled on an old predator exclusion cage at Cape Armitage (Figure 2). This sponge was observed to have grown almost 30% when the site was revisited in 2012, only two years later, demonstrating the potential of fast growth rates relative to the 1967 through 1989 period when no growth was observed.

**Figure 4:**
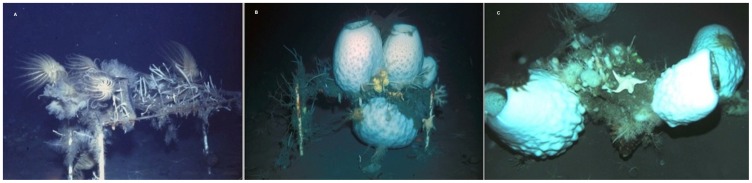
*Anoxycalyx joubini* growth on rack at New Harbor, Explorers Cove. One of the racks at Explorers Cove in (A) 1988, before *A. joubini* settlement; (B) 2004, with 4 *A. joubini*; and (C) 2010, some *A. joubini* falling off of structure, but still alive.

**Figure 5:**
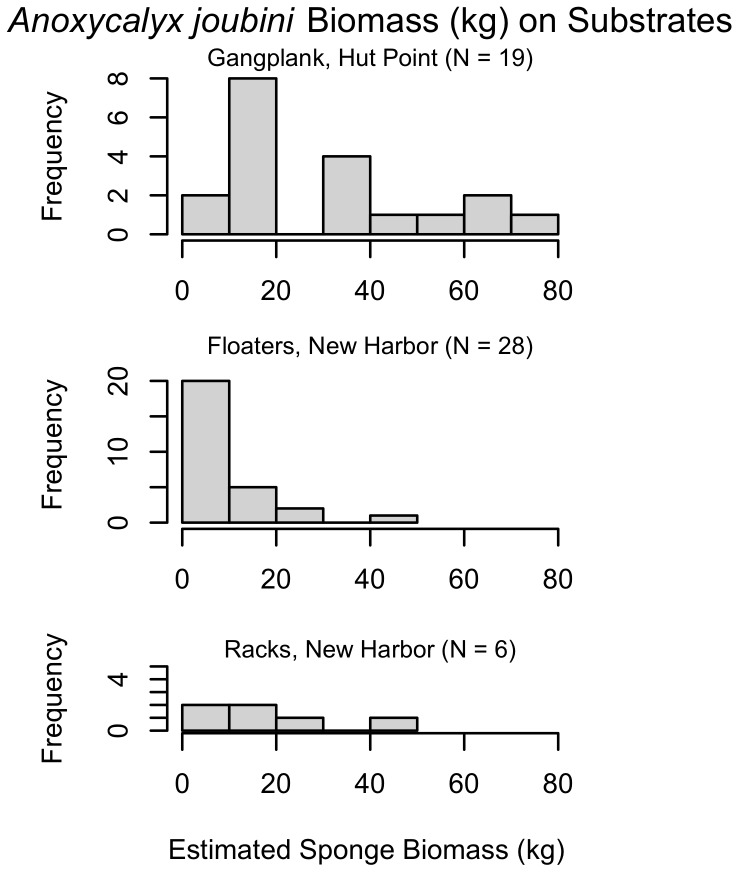
Estimated biomass (kg) of *A. joubini* on settling structures. Biomass (estimated using regression from Dayton *et al*. [8]) of *A. joubini* settled on various artificial substrates (gangplank, floater, or rack). Differences in sponge biomass between sites and substrates may be attributable to floater and rack sponge loss.

In Explorers Cove, no sponges were observed on settling surfaces from 1974 until 1989 when a few *H. balfourensis* were found on some floating surfaces and racks. In addition, one large floater that had been suspended about 15 m above the bottom in 1975 was observed to have two small hexactinellids, possibly *A. joubini* in 1989. In 2004 *A. joubini* were found on nearly all artificial substrates and by 2010, the racks (Figure 4) and floats at Explorers Cove included individual sponges over 40 kg (Figure 5). It is important to note that the Explorers Cove data are underestimates of total sponges that have recruited to the floater and rack structures, because very large sponges observed in 2004 had fallen off their substrata by 2010. Massive sponges unbalanced and tipped one floater, dumping the sponges sometime before 2010 and several other floaters had simply sunk from the weight of the sponges. In all cases, piles of *A. joubini* spicules were found on the bottom where the sponges had landed. In addition, some of the very large sponges observed on racks had become large enough to push other sponges off the structure, again reducing the total estimate of recruited individuals and biomass to the structures over this time period (Figure 4 and Figure 5). We do not know how many of these sponges either fell off their structures or sank their floater, nor do we know how they died; however, in a few cases sponges apparently were dislodged recently and appeared still alive but were infested with the amphipod, *Seba antarctica* and being consumed by *Acodontaster conspicuous*. While the exact cause of death is uncertain, it is clear that essentially all of them die after landing on the bottom. Even with many of the large sponges lost before we could measure them, the estimated mean sponge weight (kg) of *A. joubini* found on the floaters was over 13 kg and nearly 18 kg on the rack (Figure 5).

We observed a high mortality of *A. joubini*. Nearly every sponge that fell from artificial substrates to the bottom after 2004 was dead in 2010– the exceptions were 2–3 sponges that were mostly dead and two sponges estimated to be 8 kg and 17 kg that had recently sunk a float. Moreover, all seven *A. joubini* observed in the 60 m basin area at Cape Armitage during 1967–68 were dead by 1977 [16]. We marked 35 large *A. joubini* at Cape Armitage, Hut Point and Explorers Cove in 1974 and 6 of these were dead by 1977 and none were alive in 2010. In addition, between 1974 and 1975 approximately 30 transect lines were laid at depths ranging from 20 to 60 m at Cape Armitage, Hut Point and Explorers Cove to follow long-term changes. When possible, transects were started near the large, conspicuous *A. joubini* to facilitate relocation. In total, transects included 15 large *A. joubini* at Cape Armitage and 10 at Explorers Cove, and none of these sponges were found alive in 2010. We do not know the ages of any of these sponges nor do we know precisely when they died, but all 67 of them died over 43 years. Finally, photographs taken in 2012 of the big sponges on the gangplank revealed that several appear to be dying, possibly from infestations of the amphipod *S. antarctica,* suggesting a much more rapid turnover of *A. joubini* than previously assumed. We have observed platelet ice formation on *A. joubini* at Cape Armitage and Hut Point in waters less then 33 m and anchor ice has been observed to kill sponge tissue of *H. balfourensis* [15], so it might also kill patches of *A. joubini* tissue.

## Discussion

*Anoxycalyx joubini* is one of the dominant, structure-forming species in the McMurdo Sound region of Antarctica [17]. It is also the largest sponge in the Antarctic and has been considered long-lived and slow growing. Here we report a highly episodic, massive and Sound-wide recruitment event and subsequent growth spurt, which occurred mainly on artificial structures. We believe that this occurred in the early 2000s although it could have started after 1989 when we last visited the structures. We lack information on the most obvious and interesting observations: the mode of reproduction, the settlement biology, and the growth of this interesting sponge. The obvious questions relate to the explanation of the event. We have no knowledge of the actual propagules or the settlement, only recruitment to a size that can be seen and identified. There are no published descriptions of dispersal propagules of *A. joubini*, their settlement preferences, or their growth rates. We have seen very small buds that we assume are asexually produced by another hexactinellid, *R. antarctica* [8], and we have collected them in the water column in strong currents. Thus, we know that asexually produced buds can move through the water column where they could in principle be entrained and lifted by strong tidal currents [12]; however, we have not seen *R. antarctica* or any other hexactinellid beside *A. joubini* on any of our settling surfaces. To our knowledge, there is no evidence of any Antarctic hexactinellid sponge demonstrating sexual reproduction, although it has been seen elsewhere [2]. In our cases *A. joubini* propagules must have been abundant, at least around the gangplank on Ross Island and at Explorers Cove (Figure 2) where there was massive recruitment high in the water column. Given the heavy recruitment observed on artificial surfaces well above the seafloor, we suggest that swimming larvae are released episodically.

Why is the *A. joubini* recruitment predominantly on artificial surfaces? We have no data to address this interesting question, but we hypothesize that there are more predators on natural substrata and that these predators serve as a strong filter on the survivorship of the propagules as discussed by Thorson [18]. Oliver and Slattery [19] offer strong evidence of the efficiency of a micro-canopy of carnivorous invertebrates near the gangplank, and Suhr et al. [20] demonstrated that three of the most common foraminifera, especially *Astrammina rara,* consume metazoa including planktonic invertebrates in Explorers Cove. Out of this, it is reasonable to speculate that benthic predation filters settling larvae as discussed by Thorson [18].

Another obvious question relates to the fact that we saw no measurable growth of many naturally occurring *A. joubini* between 1967 and 1989, yet beginning sometime between then and 2004 (probably starting around 1998 when we last visited the eastern habitats) they exhibited tremendous growth. With the exception of two small sponges, none of the structures had any *A. joubini* in 1989. However, in 2004 these structures were photographed with very large sponges that presumably had settled after 1998, but certainly no earlier than 1990 (Figure 3 and Figure 4), and by 2010 sponges had obtained diameters ranging from 7 to 72 cm (Figure 5). Further, the estimated mass of a sponge observed on an artificial substrate at Cape Armitage in 2010 increased about 30% when it was re-photographed in 2012. Clearly, rapid growth rates are possible by *A. joubini*.

**Figure 6:**
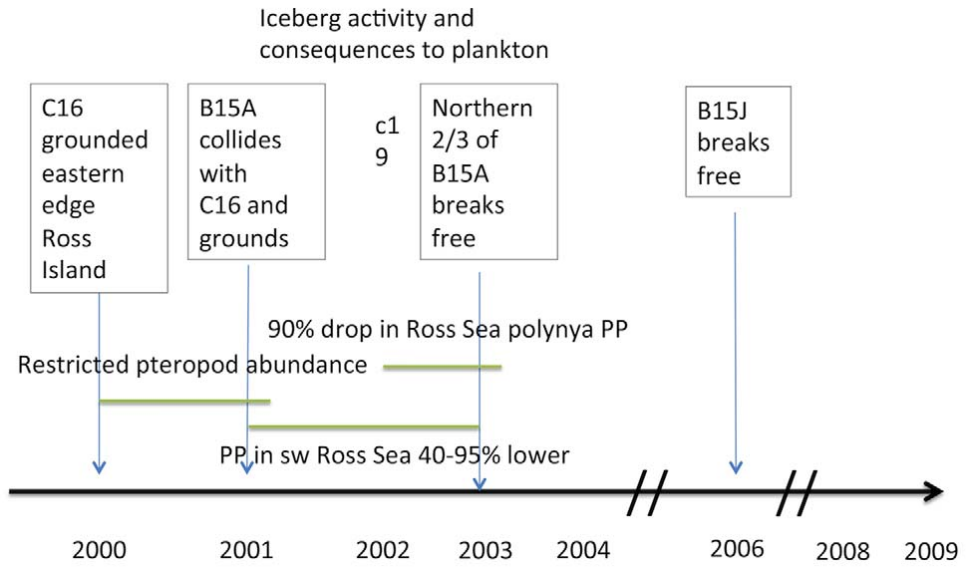
The timing of the major iceberg events in the southern Ross Sea. The combined presence of the icebergs were strongest from mid-2002 to late 2003. Figure modified from Thrush and Cummings [22].

What environmental factors were responsible for this sudden growth? The most likely correlate with the growth if not the settlement was a probable shift in plankton composition. Typically (before 2000) the transport of abundant primary production from the north [13], [21] results in a seasonal plankton bloom composed of relatively large phytoplankton (Figure 2). However, in the 2000s a series of large icebergs were grounded, blocking this transport and preventing the annual ice from breaking up and going out until 2011. The icebergs and thick sea-ice probably interfered with the advection and growth of the large phytoplankters that usually dominate in the water column [22]–[24]. The iceberg was not a single event but a series of events so the southerly flow was effectively blocked for a decade (Figure 6). Thrush and Cummings [22] and Conlan et al. [25] summarized many populations that were negatively impacted by the lack of advected primary production over this decade.

The dynamics of *A. joubini* were also correlated with this phenomenon, and we suggest that changes in the plankton [22], [25] may have resulted in a shift from large phytoplankters to tiny dinoflagellates and bacteria. Margalef [26] postulated such a relationship in water columns to result from reduced resources. Sea ice thickness and transparency affects benthic productivity and ecosystem function [27], [28]. Montes-Hugo et al. [29], described such regional changes in the Western Antarctic Peninsula suggesting a strong relationship between ice cover and the size of the phytoplankton. Orejas et al. [30] and Thurber 2007 [31] discuss the strong relationship between microplankton and Antarctic sponges. Reiswig [32] and Yahel et al. [33], working on other hexactinellid sponges, demonstrated that they retain only very small particles of bacteria and protists. As hexactinellids in general seem restricted to feeding on tiny particles [2], the shift in plankters may have offered a strong pulse of appropriate food for *A. joubini,* triggering rapid growth that was previously not observed in this species. Moreover, our observations of relatively fast growth following a shift in the food is supported by Kahn et al. [34] who report relatively fast temporal changes in the density of two deep-water hexactinellid sponge species in 4,000 m depth off Monterey, California, USA. These density shifts occur with a lag of 1–2 years following shifts in the food supply of the micro-particles they consume.

Although *A. joubini* growing on the gangplank had a broader weight distribution than the same species growing on the floaters in Explorers Cove (Figure 5), we are hesitant to attribute these differences to the site location. It is very likely that the individual sponges that fell off the racks and floaters in Explorers Cove were larger than the sponges that remained (and were measured) on these substrata. Therefore, the measurements from these two substrata at Explorers Cove could be skewed to smaller-sized individuals.

We also have preliminary but convincing evidence of *A. joubini* mortality. Although we were not able to relocate all transects in 2010 and therefore may have missed some surviving sponges, at least 67 large *A. joubini* died in the 40 years of this program with no known survivors. We have no reason to question earlier observations [8], [9] that some mortality results from predation by *A. conspicuous* and the amphipod *S. antarctica* [19]. Additionally, Cerrano et al., [35] report patches of diatoms inside *A. joubini,* but speculate that the diatoms had invaded and are detrimental to the sponges. We agree and have seen the amphipod, *S. antarctica,* eating patches of the sponge that subsequently are colonized by diatoms [9]. In 2012 we photographed considerable evidence of incipient amphipod infestation on *A. joubini* at the gangplank; however, the actual mortality sources within this study are not known and some may reflect ice formation on the sponge that kills the tissue [15] in a patchy manner, later becoming infected with *S. antarctica*. We emphasize that many of these large *A. joubini* surely do live longer, and we are only considering sponges in our localized study sites, but this is still a very high mortality rate for a species of sponge thought to be long-lived. Summarizing the *A. joubini* observations of massive recruitment and growth and rapid mortality, we suggest that this sponge has much more dynamic life history than previously suspected.

What of the other Hexactinellida in our study sites? We know that *R. antarctica* (then identified as *R. racovitzae*) grows relatively fast as this was studied in the 1970s [9]. We observed surprisingly fast growth and asexual reproduction of mature individuals and we also observed some 40 very small *R. antarctica* buds to increase their volume as much as two orders of magnitude (Table 1 in [9]). This species is by far the dominant sponge in the 25–50 m depth range at McMurdo Station [8], [9], but it is so inconspicuous that it is extremely difficult to evaluate the population patterns. Obviously it has the potential to multiply relatively quickly, yet we have no evidence of sufficient mortality to balance the reproduction and growth rates observed [9].

The other common Antarctic hexactinellid is *R. nuda/racovitzae*. This knobby, volcano-shaped sponge is smaller than *A. joubini* and remains an enigma with regard to its population dynamics and growth rate. Prior to the removal of the cages in 1977, seven *R. racovitzae* survived inside cages (two survived 9 years and 5 more survived 3 years), while 2 died inside their cages. Those survivors did not show significant growth during that time period. The mortalities may have resulted from sea star predation or infestation of *S. antarctica* [9]. Our extensive surveys in 2010 may have come across a few young *R. nuda/racovitzae* although they were not collected and we are not sure of their identification. It is interesting to note that Fallon et al. [36], report a relatively-small, 15 cm diameter specimen from the Ross Sea was approximately 440 years old. Many of the *R. racovitzae* in our area were at least a meter tall, so this species might obtain great age.

*Rossella fibulata* is a rare sponge in the McMurdo Sound area; however, two individuals settled on a rack at Explorers Cove and on a cage at Cape Armitage. It appears to grow rapidly but otherwise little is known of its biology. In any case, the four hexactinellid species in this shallow habitat certainly have different life history patterns, with the fast turn-over of *A. joubini* being the most surprising. Our observations complement those of Teixidó et al. [37] who report high frequencies of asexual reproductive strategies in three deep-water Hexactinellida in which 35% of the observed *R. nuda* were actively budding. In addition, many *R. racovitzae* exhibited reproduction by fragmentation while *R. vanhoeffeni* reproduced with bipartition. Thus, it appears that each of the Antarctic Hexactinellida species exhibits different life history biology.

In summary, these observations allow us to test and reject the prevailing notion of slow rate processes for both recruitment and growth of *A. joubini.* The population dynamics imply that *A. joubini* are fast to respond to an environmental shift, but the population increase may be relatively short (decades rather than centuries) and we need to re-evaluate ideas of slow processes and stability over century time scales. These surprising results are set in a time of climate- and fishing-related environmental changes. Certainly these results demonstrate the great importance of comprehensive, long-term data sets designed to better understand such processes.

All the photographs are available from the Scripps Archives and invertebrate collection, Scripps Institution of Oceanography, as well as “Antarctic Master Directory, via the National Antarctic Data Coordination Center.” Available: http://www.usap-data.org/. Voucher specimens collected in the 1960s were sent to the Smithsonian Oceanographic Sorting Center and the specimens seem to be lost; however, a collection of specimens is available at the Scripps Invertebrate Collections.
